# Expression of Treg/Th17 cells as well as related cytokines in patients with inflammatory bowel disease

**DOI:** 10.12669/pjms.325.10902

**Published:** 2016

**Authors:** Xianhui Geng, Jie Xue

**Affiliations:** 1 Xianhui Geng, Department of Gastroenterology, PLA 153rd Central Hospital, Zhengzhou 450042, China; 2 Jie Xue, Department of Ultrasonography, Zhengzhou People’s Hospital, Zhengzhou 450003, China

**Keywords:** Inflammatory bowel disease, Regulatory T cells, T helper 17 cells, cytokines

## Abstract

**Objective:**

To investigate the expressions of peripheral regulatory T cells (Treg) and T helper cells (Th17) as well as related cytokines in peripheral blood of patients with inflammatory bowel disease (IBD).

**Methods:**

One hundred four cases of IBD patients admitted in our hospital were selected for this study. One hundred cases of people receiving healthy physical examination were included in the control group in the corresponding period. The levels of CD4^+^CD25^+^Treg and Th17 subsets were analyzed in peripheral blood of two groups using flow cytometry. The expressions of IL-10, TGF-β1, IL-17 and IL-23 mRNA and protein were detected using real-time fluorescence quantitative PCR and ELISA.

**Results:**

Compared with the control group, the proportion of Treg in peripheral blood was decreased significantly in observation group (P<0.05), the proportion of Th17 cells was increased significantly (P<0.05), and Treg/Th17 was decreased significantly (P<0.05). Compared with the control group, the expressions of IL-10 and TGF-β1 mRNA and protein in peripheral blood of patients were significantly down-regulated in observation group, while the expressions of Th17 cytokines IL-17 and IL-23 mRNA and protein were significantly increased (P<0.05).

**Conclusion:**

The proportion of Th17 and increased cytokine level suggested the inflammatory level was higher in IBD patients. The down regulations of Treg and cytokine suggested that the immunosuppression function was down-regulated in IBD patients, and the disproportionality might be one of the mechanisms of IBD.

## INTRODUCTION

Inflammatory bowel disease (IBD) refers to a group of unknown etiological non-specific chronic inflammations involving the gastrointestinal tract. The narrowly defined IBD includes Crohn’s disease (UC) and ulcerative colitis (CD).^1^-^3^ The pathogenesis of IBD is unclear, which may be related to immune, heredity, infection and mental factors etc.^4^,^5^ Immunity, especially the relationship between intestinal immunological environment and IBD is the study focus.^6^ T helper 17 cells (Th17) is a group of newly discovered CD4^+^ positive T lymphocyte subset, mainly mediating chronic inflammation and autoimmune diseases.^7^-^9^ The present study has shown that Th17 is abnormally expressed in IBD patients. Regulatory T cells (Treg) is another kind of CD4^+^ T lymphocyte subset, mainly mediating the inhibition of autoimmunity.^10^-^12^ Th17 and Treg can be mutually transformed. Their balance plays an important role in the maintenance of intestinal immune homeostasis.^13^ The proportion of Treg/Th17 subset as well as related cytokines in peripheral blood were analyzed in this study, which might provide certain theoretical basis for the further study of the pathogenesis of IBD.

## METHODS

One hundred four cases of IBD patients admitted in our hospital were selected for this study from June 2013 to December 2015.

### Inclusion criteria

(1) All patients accorded with the clinical diagnostic criteria of IBD in “Consensus of inflammatory bowel disease diagnosis and treatment standard in China” formulated by digestion branch of Chinese Medical Association in 2007.^14^ (2) Biochemical indexes detection displayed a large number of red blood cells and white blood cells, but the bacterial culture was negative; (3) Imageological examination showed that patients underwent the intestinal double-contrast barium enema; (4) Endoscope displayed herpes-like change.

### Exclusion criteria

(1) The patients suffered from specific enteritis or a variety of infectious gastrointestinal diseases induced by bacteria; (2) Patients with autoimmune disease; (3) Women during lactation or pregnancy period; (4) Patients with psychiatric disorder. Among 104 included patients, 68 were males and 36 were females; aged 23-48 years, averagely aged 30.5±13.1 years; including 28 cases of CD and 76 cases of UC. 100 cases of receiving healthy physical examination were included in the control group in the corresponding period, including 68 males and 32 females; aged 20-51 years, averagely aged 31.5±14.1 years. The age, gender and other indicators showed no statistical difference between the two groups. This study was conducted in accordance with the declaration of Helsinki after approval from the Ethics Committee of Zhengzhou People’s Hospital. Written informed consent was obtained from all participants.

### Blood sample collection

The venous sampling was performed in the morning from the fasting patients in the observation group. The same method was performed on the volunteers in the control group. After the blood was anticoagulated, red blood cell lysis (*Sigma*, *St*. *Louis*, *MO*, *USA*) was used to remove red blood cells. The white blood cells were resuspended in PBS solution for the following experiments.

### Flow cytometry analysis

The white blood cells were washed with PBS twice and the cell counting was performed. The cells were adjusted to 1*10^6^/ml and divided into two tubes for one patient. One tube was labeled with Th17 and the other tube was labelled with Treg. The labelling method of Th17 was as the following: the cells were centrifuged by 1000 rpm for 5 min, re-suspended in 4% PBS containing paraformaldehyde, fixed for 2 h at room temperature, washed for 3 times with PBS, five minutes/time. Then the membrane rupture was added at room temperature and stood for four minutes, centrifuged by 1000 rpm for five minutes and suspended using 100 μl PBS. 2 μl CD4-FITC and IL-17-PE labeled antibody (eBioscience, SanDiego, CA, USA) was added in each tube, incubated for 1 h on ice at 4°C, centrifuged by 1000 rpm for 5 min, washed for three times with PBS, five minutes /time. Finally, the cells were re-suspended in 400 μl PBS solution for flow cytometry analysis (BD Biosciences, New Jersey, USA). Treg labeling method: the cells were centrifuged by 1000 rpm/min for 5min and resuspended in 100 μl PBS solution. 2 μl CD4-FITC and CD25-PE labeled antibody (eBioscience, SanDiego, CA, USA) was added in each tube, incubated on ice at 4°C for 1 h, centrifuged by 1000 rpm for 5min, washed 3 times with PBS, 5 min/time. Finally the cells resuspended in 400 μl PBS for flow cytometry analysis (BD Biosciences, New Jersey, USA).

### Real-time fluorescent quantitative PCR

The white blood cells of the patients or volunteers were used to extract total RNA according to the RNA extraction kit (TaKaRa, Dalian, China). According to the mRNA sequences of IL-17, IL-23, IL-10, TGF-β1 provided by the GeneBank, the primers were designed as the following: IL-17-F: 5’-TGTCCACCATGTGGCCTAAGAG-3’, IL-17-R: 5’-GTCCGAAATGAGGCTGTCTTTGA-3’; IL-23-F: 5’-ACAACTGAGGGAACCAAACCA-3’, IL-23-R: 5’-GCATGATGAATTGTAGTAGCGG-3’; TGF-β1-F: 5’-AGCGACTCGCCAGAGTGGTTA-3’, TGF-β1-R: 5’-GCAGTGTGTTATCCCTGCTGTCA-3’; IL-10-F: 5’-TGCCTTCAGTCAACTGAAGAC-3’, IL-10-R: 5’-AAACTCATTCATGGCTTGTAC-3’; β-Actin-F: 5’-GCGGGAAATCGTGCGTGAC-3’, β-Actin-R: CGTCATACTCCTGCTTGCTG-3’. The primers were diluted to 10 µmmol•L^-1^. The primer specificity and annealing temperature were optimized. Then the following reaction system was prepared: 2*SYBR Green universal qPCR Master Mix (TaKaRa, Dalian, China) 10 µl, upstream/downstream primers (10 µmmol•L-1) 0.6 µl, 1:100 diluted cDNA 8.8 µl. The total reaction volume was 20 µl. Then the reaction mixture was thrown into the bottom of the tube by 1500 rpm/min centrifugation. PCR was conducted according to the following reaction conditions: predenaturation at 95°C for 30 s; denaturation at 95°C for 3 s; annealing at 60°C for 30 s; The solubility curve was established. Finally, the data were directly read from the fluorescent quantitative PCR instrument (Bio-Rad, Hercules, CA, USA).

### ELISA analysis

The serum of the subjects was diluted into 1:100. The levels of IL-17, IL-23, IL-10 and TGF-β1 were detected strictly in accordance with the instruction book of ELISA kit (R&D Systems Inc, Minneapolis, MN, USA). Three complex holes were set for each sample and standard. The OD values were measured at 492nm using the microplate reader (Thermo Scientific, Rockford, IL, USA).

### Statistical analysis

All data were analyzed using SPSS13.0 statistical software (SPSS Inc, Chicago, IL, USA). The measurement data were expressed using X±S and analyzed with t test. P<0.05 indicated that the difference was statistically significant.

## RESULTS

### Comparison of Th17 and Treg proportions between two groups

**Fig.1:**
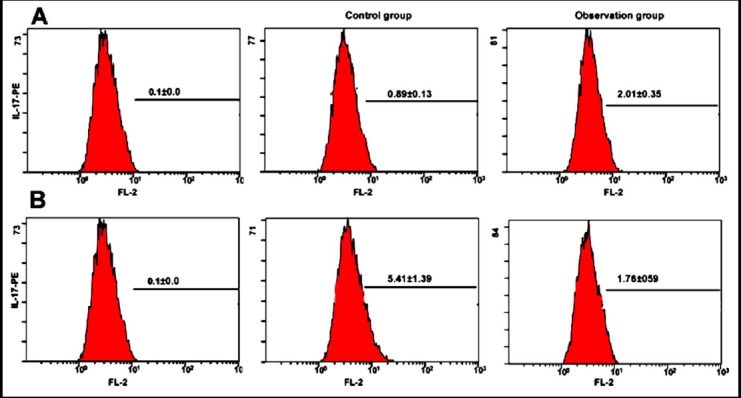
Comparison of Th17 and Treg cells in two groups. A. Comparison the proportion of Th17 cells in two groups; B. Comparison the proportion of Treg cells in two groups.

Compared with the control group, the proportion of CD4^+^IL-17^+^ positive cells in peripheral blood of patients in the observation group was increased significantly, while the proportion of CD4^+^CD25^+^ positive Treg was significantly down-regulated (P<0.05) (Fig.1).

### Comparison of Th17 and Treg mRNA levels between two groups

**Fig.2:**
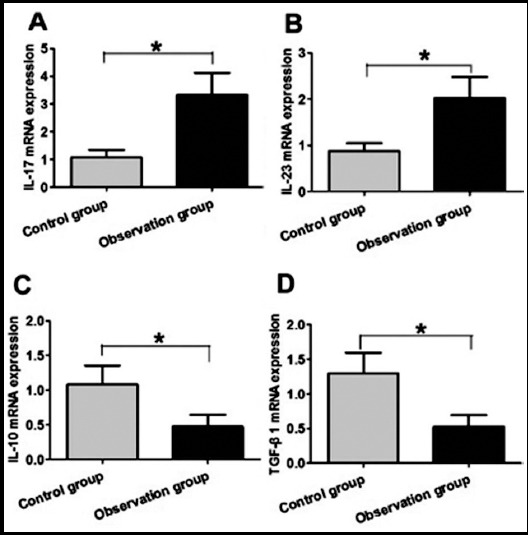
Comparison of Th17 and Treg mRNA levels in two groups. A. IL-17 mRNA levels in two groups; B. IL-23 mRNA levels in two groups; C. IL-10 mRNA levels in two groups; D. TGF-β1 mRNA levels in two groups.

As shown in Fig.2, compared with the control group, the mRNA levels of IL-17 and IL-23 in Th17 cells were significantly increased, the difference was statistically significant (P<0.05). Compared with the control group, the mRNA levels of Treg IL-10 and TGF-β1 in Treg cells were significantly decreased, the difference was statistically significant (P<0.05).

### Comparison of Th17 and Treg protein levels between two groups

**Fig.3:**
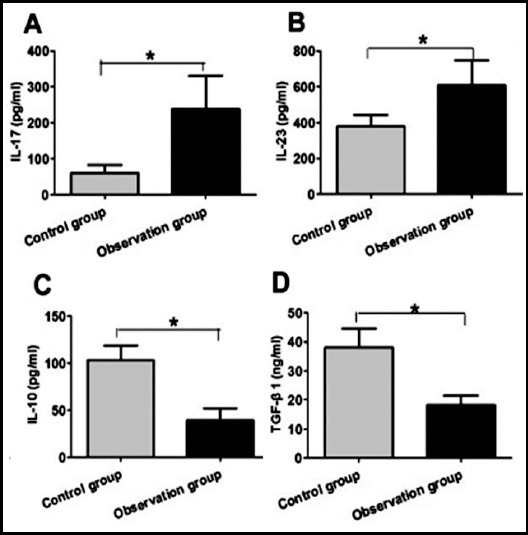
Comparison of Th17 and Treg protein levels in two groups. A. IL-17 protein levels in two groups; B. IL-23 protein levels in two groups; C. IL-10 protein levels in two groups; D. TGF-β1 protein levels in two groups.

As shown in Fig.3, compared with the control group, the protein levels of IL-17 and IL-23 in Th17 cells were significantly increased. And the difference was statistically significant (P<0.05). Compared with the control group, the protein levels of IL-10 and TGF-β1 in Treg cells were significantly decreased. And the difference was statistically significant (P<0.05).

## DISCUSSION

The pathogenesis of inflammatory bowel disease (IBD) is still a problem to be solved. Many studies have found that IBD is closely related to the patients’ body immunity especially intestinal immune microenvironment.^15^ Previous studies have showed that IBD was an allergic inflammation induced by the imbalance of Th1/Th2 immune response, dominating with intestinal epithelial Th1 immune response.^16^ However in recent years, with in-depth study of CD4^+^T lymphocytes, the result found that the initial CD4^+^T cells could differentiate into different cells induced by the different cytokines. Specifically, the initial CD4^+^T cells could differentiate into Th17 under the mutual effect between TGF-β and IL-6. This kind of cells could secrete IL-17, IL-23, IL-21 and other cytokines, which was mainly involved in inflammation and autoimmune diseases.^17^,^18^ The initial CD4^+^ cells could differentiate into Treg under the simple stimulation of TGF-β. This kind of cells mainly inhibited inflammatory reaction.^17^,^19^ Th17 and Treg could promote or inhibit the mutual effect of inflammation. Their dynamic balance was a key factor in many inflammatory or autoimmune diseases.

The essence of IBD is a kind of chronic inflammatory disease. Report from animal level has showed that the number of CD4+ cells expressing IL-17 in the intestinal tract and lymphoid organs in mice were 20 times of the normal control mice, indicating that Th17 proportion in the intestinal tract or lymphoid organs in mice was up-regulated significantly. This result has suggested that Th17 might participate in the occurrence of IBD.^20^ Therefore, the changes of Th17 and Treg with antagonistic action were analyzed in peripheral blood of IBD patients. Study results showed that compared with the normal volunteers, the proportion of Th17 cells in peripheral blood of IBD patients was significantly increased, while the proportion of Treg was significantly decreased, indicating that the ratio of Th17/Treg lost the balance. The proportion of Th17 was increased and the proportion of Treg cells was decreased, indicating that patients had obvious inflammation, which was also consistent with patients’ clinical symptoms.

Th17 cells could secrete IL-17, IL-23, IL-21 and other cytokines, promote inflammation; While Treg could secrete IL-10, TGF-β and other cytokines, inhibit inflammation.^21^ Previous results showed that the proportion of Th17 was increased and the proportion of Treg was decreased. However, their functions relied on the cytokines. Therefore, the levels of Th17 and Treg secreting cytokines were analyzed using real-time fluorescent quantitative PCR and ELISA methods. The results of the study showed that compared with the healthy control group, the levels of IL-17, IL-23 and other cytokines in the peripheral blood of IBD patients were significantly increased, while the levels of IL-17, IL-23 and other cytokines were significantly down-regulated, which was consistent with the changes of Th17 and Treg proportions. IBD patients had TH17/Treg and cytokine imbalances, further suggesting that Th17/Treg imbalance resulted in IBD.

In short, the levels of Th17 cells and cytokine were increased significantly in IBD patients, while the levels of Treg and cytokine were significantly down-regulated. Th17/Treg imbalance was one of the reasons of IBD.
